# A reliable set of reference genes to normalize oxygen-dependent cytoglobin gene expression levels in melanoma

**DOI:** 10.1038/s41598-021-90284-6

**Published:** 2021-05-25

**Authors:** Joey De Backer, Darko Maric, Matthias Bosman, Sylvia Dewilde, David Hoogewijs

**Affiliations:** 1 Research Group PPES, Department of Biomedical Sciences, University of Antwerp, Antwerp, Belgium; 2 Section of Medicine, Department of Endocrinology, Metabolism and Cardiovascular System, University of Fribourg, Fribourg, Switzerland

**Keywords:** Gene expression analysis, Reverse transcription polymerase chain reaction, Molecular biology

## Abstract

Cytoglobin (CYGB) is a ubiquitously expressed protein with a protective role against oxidative stress, fibrosis and tumor growth, shown to be transcriptionally regulated under hypoxic conditions. Hypoxia-inducible *CYGB* expression is observed in several cancer cell lines and particularly in various melanoma-derived cell lines. However, reliable detection of hypoxia-inducible mRNA levels by qPCR depends on the critical choice of suitable reference genes for accurate normalization. Limited evidence exists to support selection of the commonly used reference genes in hypoxic models of melanoma. This study aimed to select the optimal reference genes to study *CYGB* expression levels in melanoma cell lines exposed to hypoxic conditions (0.2% O_2_) and to the HIF prolyl hydroxylase inhibitor roxadustat (FG-4592). The expression levels of candidate genes were assessed by qPCR and the stability of genes was evaluated using the geNorm and NormFinder algorithms. Our results display that *B2M* and *YWHAZ* represent the most optimal reference genes to reliably quantify hypoxia-inducible *CYGB* expression in melanoma cell lines. We further validate hypoxia-inducible CYGB expression on protein level and by using *CYGB* promoter-driven luciferase reporter assays in melanoma cell lines.

## Introduction

Over the last few decades, gene expression analysis has become increasingly more important, as the understanding of gene expression patterns can reveal complex regulatory networks involved in disease initiation or progression^1^. Nowadays, the method of choice for individual gene expression analysis is real-time quantitative PCR (qPCR). qPCR is characterized by a high sensitivity and sequence-specificity, and a broad dynamic range^2^. An inherent drawback associated with the sensitivity is the need for an accurate way of normalization and standardization. Variations in the amount of starting material, RNA extraction, and enzyme efficiencies are inherently associated with the multistep qPCR workflow^3^. Consequently, obtaining reliable gene expression patterns require an accurate normalization strategy.

Currently, the method of choice for (data) normalization is through the use of internal reference genes and by the analysis of relative gene expression using the 2^−ΔCt^ method^4,5^. The most commonly used reference genes are constitutive genes that regulate basic ubiquitous cellular functions^6^. It has been shown however that the expression of these genes is not stable under various experimental conditions^6–8^. Hypoxic conditions in particular have recently been shown to pose a hurdle for gene expression studies. For example, glyceraldehyde-3-phosphate dehydrogenase (*GAPDH*), β-actin (*ACTB*), and β-tubulin (*TUBB*), three of the most commonly used reference genes, were shown to be transcriptionally modulated upon hypoxia in specific cell types^1,7,9,10^, possibly leading to misinterpretation of changes in target gene expression. Therefore, gene expression should always be normalized with an appropriate, i.e. neither influenced by experimental conditions nor differently regulated in the samples being studied, reference gene^11^. As identifying such a gene might be rather difficult, normalization by geometric averaging of multiple internal reference genes is currently considered the most appropriate and universally applicable approach in the evaluation of qPCR-based gene expression^3,12,13^. The statistical software algorithm geNorm represents a well-established tool for the identification of the most stably expressed genes from a set of candidate control genes. The method also allows the determination of the optimal number of genes required for reliable normalization of qPCR generated gene expression data.

Hypoxia is a key microenvironmental factor during the initiation, progression, and propagation of cancer^14,15^. In solid tumors, the intensive proliferation of cancer cells combined with the structural abnormalities of the tumor vasculature results in the delivery of suboptimal concentrations of oxygen and other nutrients to cancer cells, creating a hypoxic milieu^14,16^. As a survival strategy, major adaptive pathways are activated in hypoxic cancer cells and cells undergo reprogramming of the transcriptional activity towards more aggressive and therapy resistant phenotypes^17^. In melanoma hypoxia also plays a crucial role and contributes to radiotherapy resistance^16^. Melanoma arises from pigment-producing melanocytes located in the basal layer of the epidermis of the skin. The skin is a mildly hypoxic environment and oxygen levels are sufficiently low enough to allow stabilization of the hypoxia-inducible factor α (HIF-α) subunit, thereby increasing the expression of established HIF target genes such as carbonic anhydrase IX (*CAIX*), glucose transporter-1 (*GLUT1*) and prolyl hydroxylase domain-2 (*PHD2*)^18–20^. Furthermore, a hypoxic microenvironment contributes to the oncogenic transformation of melanocytes to melanoma and plays a pivotal role in epithelial-to-mesenchymal transition (EMT), enabling metastasis^16^. Hence, investigating the genetic alterations that contribute to melanoma initiation and progression under hypoxic conditions is essential for a better understanding of overall cellular responses, which can form the basis for novel therapeutic targets.

Cytoglobin (CYGB) is a ubiquitously expressed hexacoordinated globin recently found to be highly enriched in melanocytes, and frequently downregulated during melanomagenesis^21^. Fujita and colleagues suggested that reduced CYGB expression is implicated into the transition from melanocytes (high CYGB content) to melanoma (low CYGB content)^21^. Although the mechanism remains enigmatic, CYGB is thought to play a role in the cellular response towards oxidative stress^22–25^. Response elements for HIF-1, AP-1, and NFAT have been located within the *CYGB* promoter, all of which are sensitive to hypoxia^26^, and hypoxia-dependent regulation of CYGB mRNA levels was observed in various cell types and tissues^27–30^.

In this study we selected and validated the most appropriate reference genes for analysis of *CYGB* gene expression in two melanoma cell lines (A375 and Malme-3M) under hypoxic conditions using geNorm and NormFinder algorithms. To validate the selected internal controls for the analysis of *CYGB* expression, we compared the expression of eight candidate reference genes under normoxic and hypoxic conditions as well as upon treatment with the HIF prolyl hydroxylase domain (PHD) inhibitor roxadustat (FG-4592). The presented approach can be applied to accurately normalize expression of any hypoxia-induced gene in these and likely other melanoma cell lines.

## Results

### B2M and YWHAZ are optimal reference genes for normalization of gene expression data under hypoxic conditions by real-time qPCR

To investigate the stability of eight of the most commonly used reference genes from different functional classes as recommended by Vandesompele and colleagues^12^ (*ACTB*, *UBC*, *HMBS*, *SDHA*, *HPRT1*, *TBP*, *B2M* and *YHWAZ*) within a hypoxic setting we set up an experiment containing two melanoma cell lines expressing high and moderately high endogenous CYGB levels, Malme-3M and A375, incubated under either normoxic or hypoxic conditions for 24 h. Additionally, cells were treated with the PHD inhibitor roxadustat (FG-4592) for 24 h. Data were collected using RNA from three replicate A375 and Malme-3M cultures and three independent real-time qPCR experiments were performed. In each experiment the expression levels of the candidate reference genes were measured in duplicate in eight different samples.

To identify the most stable reference genes we employed the geNorm algorithm. In Table 1, each candidate reference gene was ranked according to their stability measure value (*M*) in every biological replicate. The stepwise elimination of genes with the highest *M* value results in the ranking of the selected genes according to their expression stability with the two most stable genes ranked equally. For all three replicates, *UBC*, *TBP*, *B2M* and *YWHAZ* displayed a low degree of average expression variation in A375 and Malme-3M cells between the tested conditions, indicating that these reference genes might be optimal candidates for calculation of the normalization factor. Notably, NormFinder, an independent algorithm to assess the stability of reference genes^31^, displayed very comparable results, with *B2M* and *YWHAZ* consistently among the 3 most stable reference genes in all 3 independent replicates (Table 2).

**Table 1:** Ranking of candidate reference genes in order of their average expression variation, decreasing from top to bottom.

Replicate 1	Replicate 2	Replicate 3
B2M (0.348)	B2M (0.307)	YHWAZ (0.197)
YWHAZ (0.348)	YHWAZ (0.307)	ACTB (0.197)
TBP (0.402)	TBP (0.356)	B2M (0.306)
UBC (0.454)	UBC (0.400)	TBP (0.361)
HPRT-1 (0.618)	SDHA (0.548)	UBC (0.436)
SDHA (0.763)	HPRT-1 (0.672)	SDHA (0.520)
HMBS (2.08)	HMBS (2.03)	HMBS (0.628)
ACTB (4.56)	ACTB (4.26)	HPRT-1 (0.693)

Average expression stability values (M) are shown between brackets.

**Table 2:** Reference gene stability.

Rank	Replicate 1		Replicate 2		Replicate 3	
	GeNorm	NormFinder	GeNorm	NormFinder	GeNorm	NormFinder
1	**B2M**	**B2M**	**B2M**	**YWHAZ**	**YHWAZ**	**B2M**
2	**YHWAZ**	TBP	**YHWAZ**	HPRT-1	ACTB	TBP
3	TBP	**YWHAZ**	TBP	**B2M**	**B2M**	**YWHAZ**
4	UBC	ACTB	UBC	TBP	TBP	ACTB
5	HPRT-1	UBC	SDHA	UBC	UBC	UBC
6	SDHA	HMBS	HPRT-1	SDHA	SDHA	HMBS
7	HMBS	HPRT-1	HMBS	HMBS	HMBS	HPRT-1
8	ACTB	SDHA	ACTB	ACTB	HPRT-1	SDHA

Ranking of selected reference genes based on stability.

Similar to GeNorm, NormFinder is a mathematical algorithm used to identify the best normalization gene according to their expression stability (*M*)^31^. Two consistent most stable references genes are labeled in bold.

**Figure 1:**
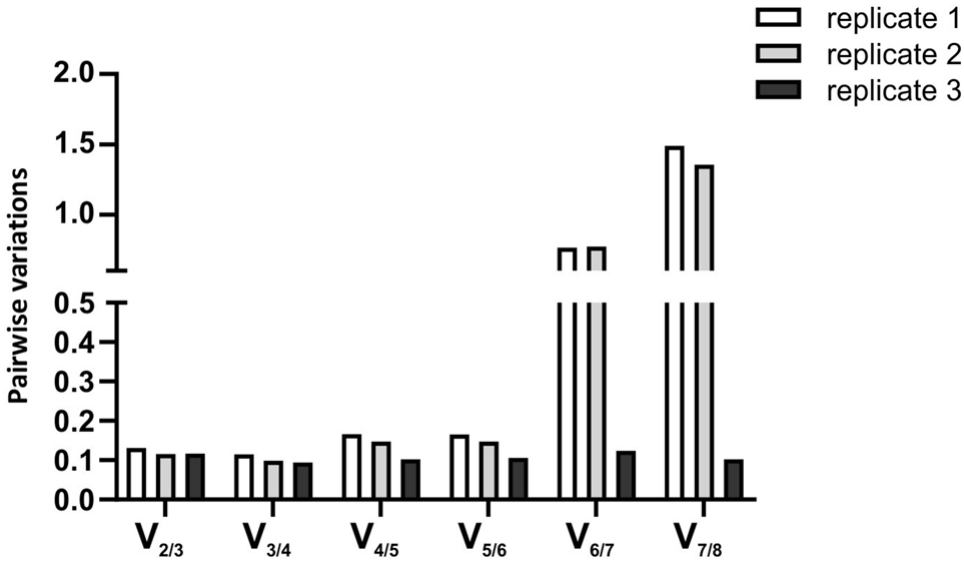
Pairwise variations (V_n/n+1_) for all three replicate experiments. A large variation between two sequential normalization factors means that the added gene has a significant effect and should be preferably included for calculation of the normalization factor. Addition of a 3^rd^ reference gene does not result in further improvement to the normalization factor in each of the three replicates.

In order to determine the number of optimal candidate reference genes that should be used in the normalization process, the pairwise variation *V*_*n*/*n*+1_ was calculated between the two sequential normalization factors (NF_*n*_ and NF_*n*+1_) for all samples, using geNorm. As recommended by Vandesompele et al.^12^ a cut-off value of 0.15 was used, below which the inclusion of an additional reference gene does not result in a substantial improvement of normalization. According to this criterion, no major improvement in normalization factor calculation was visible when three (or more) genes were included, indicating that two reference genes are sufficient for the normalization process (Fig. 1). More specifically, our results illustrated that *B2M* and *YWHAZ* are the most optimal reference genes for normalization of qPCR-based relative expression levels within a hypoxia-based experimental setup involving A375 and Malme-3M cells.

### CYGB mRNA expression levels are hypoxia-inducible in A375, but not in Malme-3M

We next investigated the hypoxia-inducible regulation of *CYGB* in A375 and Malme-3M cells and determined CYGB mRNA levels as well as hypoxia-responsive control gene expression levels (*CAIX*, *GLUT1* and *PHD2*) after 24 h of hypoxia (0.2% O_2_) and upon roxadustat treatment in A375 and Malme-3M cells. A normalization factor based on the geometric mean of *B2M* and *YWHAZ* expression level was employed to analyze their relative expression level.

**Figure 2:**
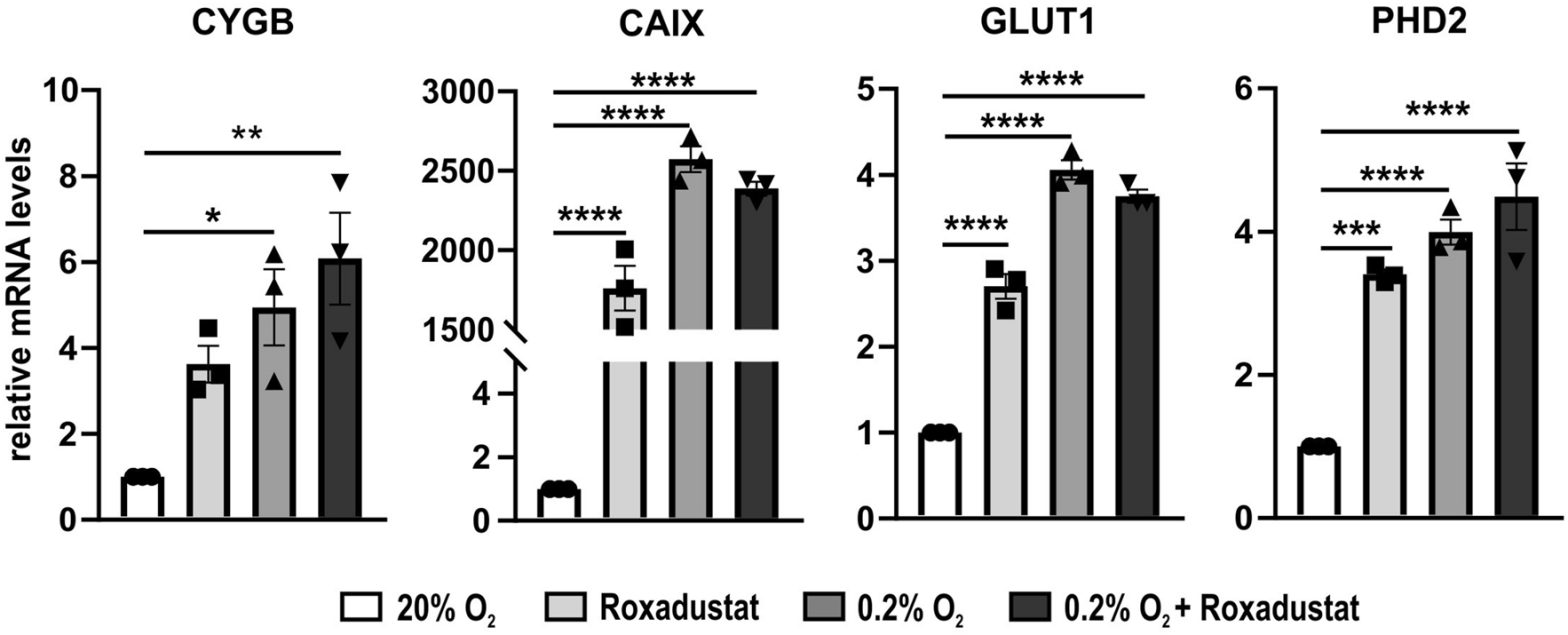
Gene expression after 24 h hypoxia or PHD inhibitor treatment in A375. Average fold change expression of three hypoxia control genes (*CAIX*, *GLUT1*, and *PHD2*) and *CYGB*, compared to the normoxic control (set as 1), after 24 h of roxadustat (100 µM), hypoxia (0.2% O_2_), and combined hypoxia and roxadustat (100 µM). qPCR values were normalized to *B2M* and *YWHAZ* (mean ± S.E.M; n = 3). Individual values of replicates are depicted as black dots. One-way ANOVA (*p ≤ 0.05; **p ≤ 0.01; ***p ≤ 0.001; ****p ≤ 0.0001).

Our results showed that in A375 expression levels of *CAIX*, *GLUT1*, *PHD2* and *CYGB* are significantly upregulated under hypoxic conditions, incubation with roxadustat (100 µM) and combined treatment of roxadustat (100 µM) and hypoxia (Fig. 2). Both hypoxia alone and the combination with roxadustat display a very similar response in the fold change expression of *CAIX*, *GLUT1*, and *PHD2*, whereas roxadustat by itself induces a lower, yet still highly significant, increase in control gene expression. Although *CYGB* expression is clearly upregulated under every experimental condition, significant regulation is only observed under hypoxic conditions and upon roxadustat treatment in the presence of hypoxic conditions.

**Figure 3:**
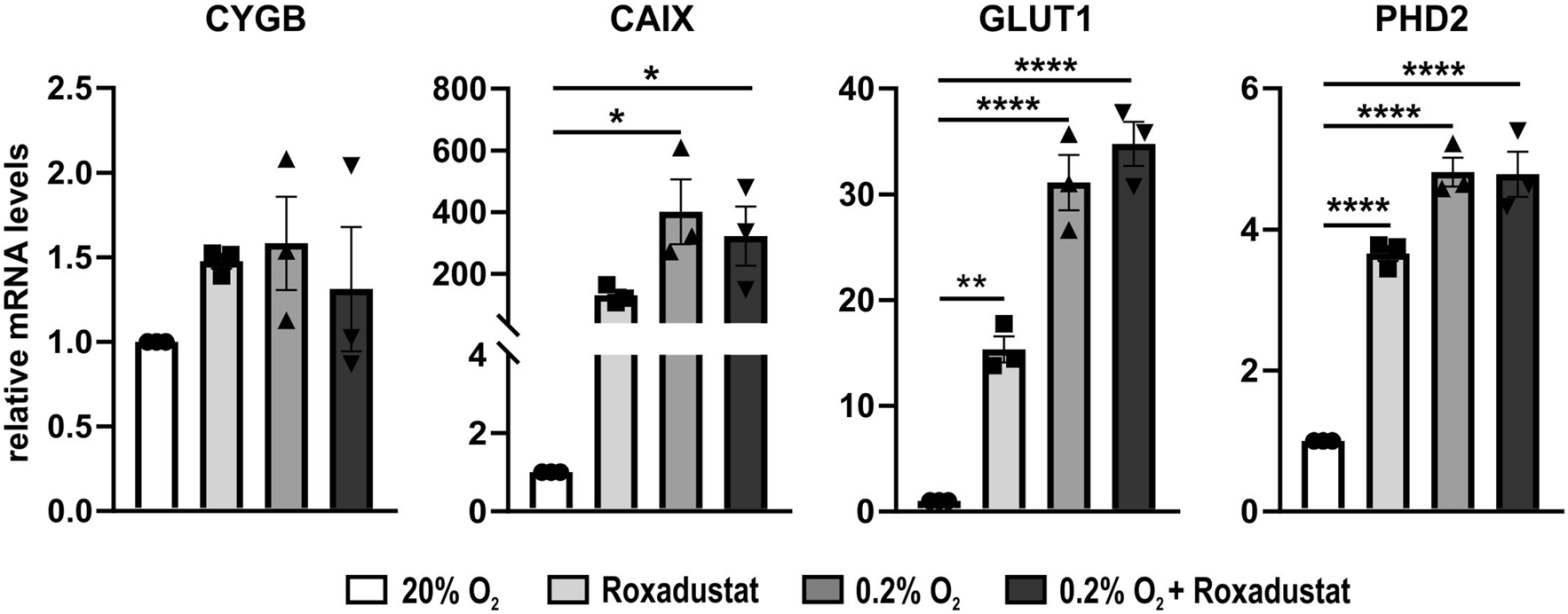
Gene expression after 24 h hypoxia or PHD inhibitor treatment in Malme-3M. Average fold change expression of three hypoxia control genes (*CAIX*, *GLUT1*, and *PHD2*) and *CYGB*, compared to the normoxic control (set as 1), after 24 h of roxadustat (100 µM), hypoxia (0.2% O_2_), and combined hypoxia and roxadustat (100 µM). qPCR values were normalized to *B2M* and *YWHAZ* (mean ± S.E.M; n = 3). Individual values of replicates are depicted as black dots. One-way ANOVA (*p ≤ 0.05; **p ≤ 0.01; ****p ≤ 0.0001).

In Malme-3M similar observations could be made (Fig. 3). *GLUT1* and *PHD2* were significantly induced upon treatment with roxadustat, hypoxic conditions, and the combination of both, whereas *CAIX* was only found to be significantly upregulated in hypoxia and the combination of hypoxia and roxadustat. Yet, a clear response in *CAIX* expression could be observed throughout all conditions. Although not statistically significant, a very slight upregulation of *CYGB* expression was detected under all conditions.

**Figure 4:**
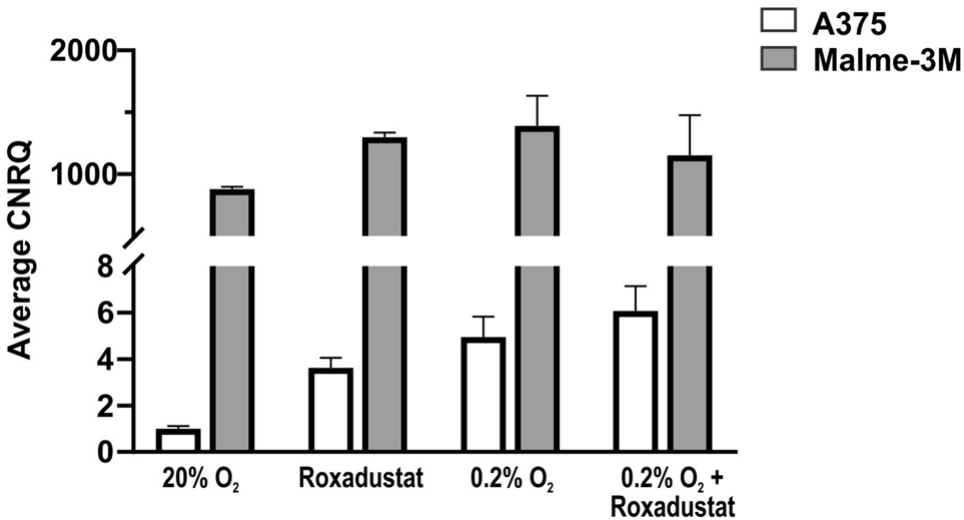
Comparison of *CYGB* expression levels in A375 and Malme-3M cells. Average *CYGB* expression, compared to the normoxic A375 control (set as 1), after 24 h of roxadustat (100 µM), hypoxia (0.2% O_2_), and hypoxia and roxadustat (100 µM). qPCR values were normalized to *B2M* and *YWHAZ* (mean ± S.E.M; n = 3). Calibrated normalized relative quantities (CNRQ) represent the relative quantity between different samples for a given target gene (i.e. *CYGB*)^52^.

Comparison of absolute *CYGB* expression values (i.e. C_t_ values) between the two melanoma cell lines Malme-3M and A375 (Fig. 4), indicated that Malme-3M cells contain substantially higher endogenous *CYGB* expression than A375, with an average expression value for Malme-3M (under normoxic conditions) more than 800 times higher as compared to A375.

### Hypoxia-dependent regulation of CYGB protein levels in A375 cells

**Figure 5:**
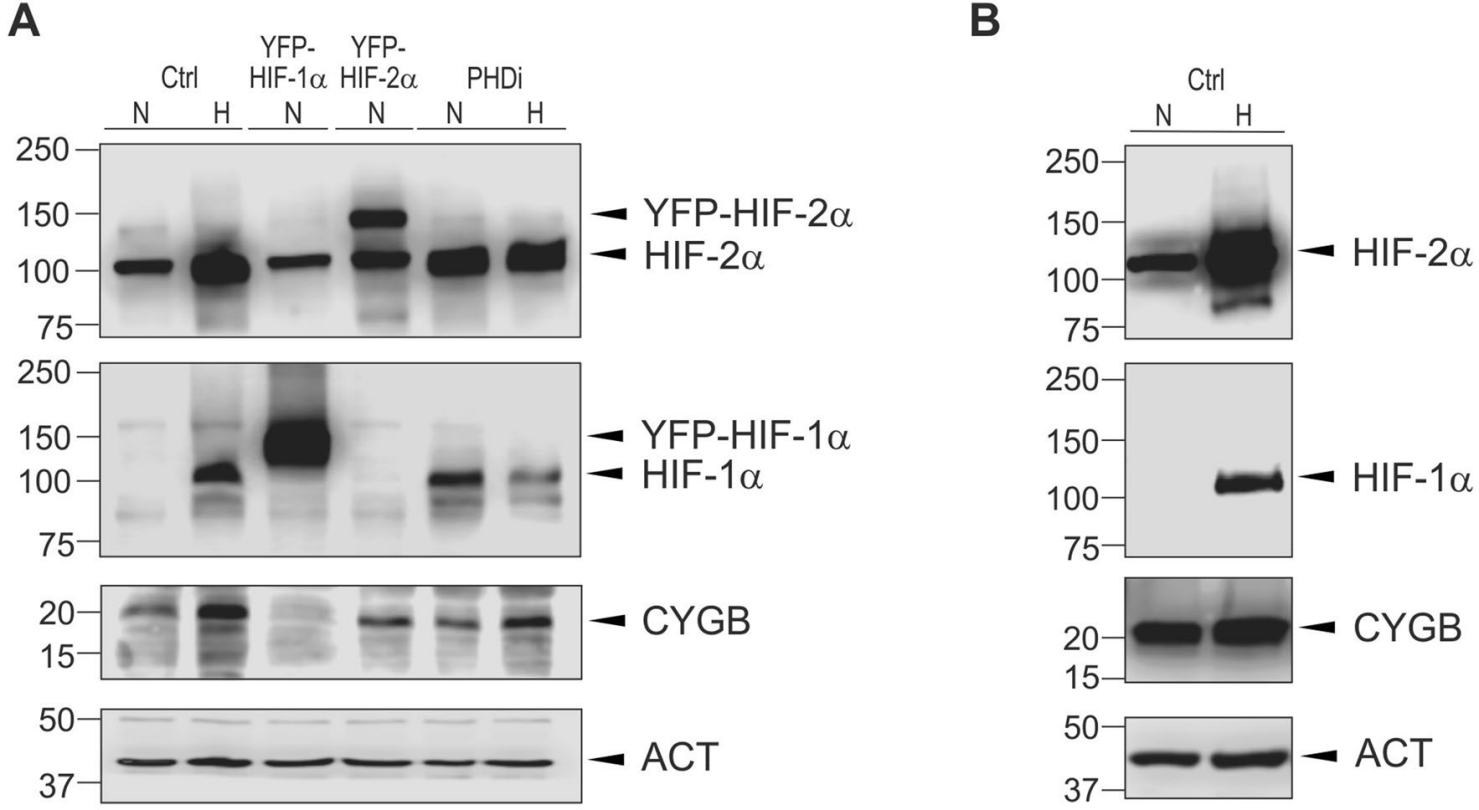
CYGB protein expression is upregulated under hypoxic conditions or in presence of PHD inhibitor. Representative immunoblots of CYGB in A375 (**A**) and Malme-3M (**B**) cells after 48 h under normoxic (N) or hypoxic (H) (0.1% O_2_) conditions, in the presence of overexpressed YFP-HIF-1α or YFP-HIF-2α (24 h), and upon treatment with 4 mM PHD inhibitor (PHDi) (24 h). HIF-1α and HIF-2α were revealed using a mouse monoclonal anti-HIF-1α or a rabbit monoclonal anti-HIF-2α antibody, respectively. CYGB was detected with a rabbit polyclonal anti-CYGB antibody. β-actin (ACT) was used as a loading control and revealed using a rabbit monoclonal anti-β-actin antibody.

Subsequently we assessed if hypoxia-inducible regulation of *CYGB* on mRNA level, could be also observed on protein level. Immunoblotting results confirmed that CYGB is strongly upregulated under hypoxic conditions (0.1% O_2_) (Fig. 5A). Interestingly, this upregulation in A375 cells is most likely HIF-2α-dependent, as no increase was observed under HIF-1α overexpression conditions. Moreover, in presence of the PHD inhibitor, we could detect a clear upregulation both under normoxic and hypoxic conditions (Fig. 5A; Supplemental Fig. S1). Consistent with absolute mRNA levels Malme-3M cells exhibited higher CYGB protein levels than A375 cells, but no regulation could be observed under hypoxic conditions (Fig. 5B).

**Figure 6:**
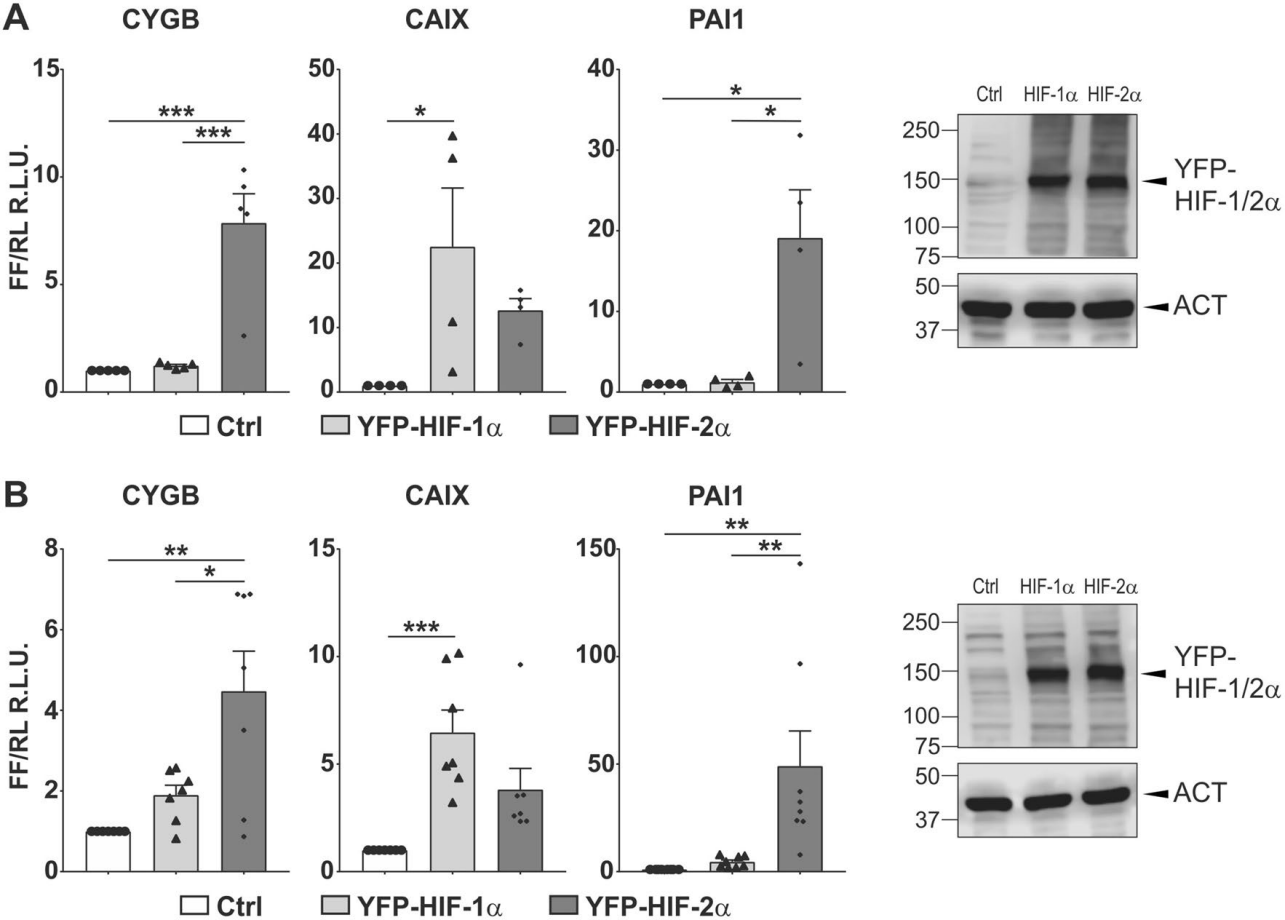
Reporter gene assays demonstrate HIF-2α-dependent induction of *CYGB* promoter-driven luciferase activity in A375 and Hep3B. A375 cells (**A**) and Hep3B cells (**B**) were transfected with *CYGB* promoter constructs and HIF-1α or HIF-2α isoform overexpression plasmids, as indicated. *CAIX* and *PAI* promoter constructs served as HIF-1α and HIF-2α control genes, respectively. For each cell line equal overexpression levels of YFP-HIF-1α and YFP-HIF-2α were detected by immunoblotting with a GFP antibody. Luciferase activity is reported as the induction compared to the control (Ctrl) and represents the ratio of firefly (FF) to *Renilla* (RL) relative light units (R.L.U.). Each column represents the mean ± SEM of four to eight different experiments performed in duplicate. One-way ANOVA (*p ≤ 0.05; **p ≤ 0.01; ***p ≤ 0.001).

To obtain additional independent support of HIF-α dependent regulation of *CYGB* we employed reporter assays using a *CYGB* promoter-driven luciferase gene*. CAIX* and *PAI1* were used as HIF-1 and HIF-2 isoform target controls, respectively (Fig. 6A). Consistent with established HIF-α isoform dependency *CAIX* promoter-driven luciferase activity was more induced upon HIF-1α overexpression whereas *PAI1* promoter-driven luciferase activity was more inducible upon HIF-2α overexpression. Immunoblotting further validated equal overexpression levels of HIF-1α and HIF-2α. Our results confirmed an eightfold induction of *CYGB* promoter-dependent luciferase activity that was only detectable upon HIF-2α overexpression, whereas HIF-1α had no effect (Fig. 6A). Finally, we validated these reporter gene assays in a non-melanoma cancer line and used Hep3B hepatocarcinomatous cells, in which *CYGB* mRNA levels were shown to be strongly induced under hypoxic conditions (Supplemental Fig. S2). Our data in Hep3B cells display a similar trend as in A375 cells with mostly HIF-2α dependent regulation, even though a moderate induction could be observed under HIF-1α overexpression conditions as well (Fig. 6B).

Taken together our results indicate that in A375 cells hypoxia-inducible *CYGB* mRNA regulation is also observed on protein level and by promoter-driven reporter gene assays, and is HIF-2α dependent under overexpression conditions.

## Discussion

Reliable qPCR results require accurate normalization based on validated stably expressed reference genes. Several reports have underlined that gene expression analyses in hypoxic cancer cells have disregarded the proper validation of the used reference genes, leading to reduced reproducibility among investigations in different laboratories^6,7^. It is now established that the stability of possible reference genes should be assessed for each cell line/tissue and experimental condition to avoid false interpretations^1,6,32^. This prompted us to undertake a comprehensive analysis of a panel of potential reference genes in two melanoma cell lines cultured under various experimental hypoxia conditions. Specifically, we included the HIF prolyl hydroxylase inhibitor roxadustat (FG-4592) as a hypoxia mimetic. Roxadustat represents an oxoglutarate analogue which was shown to increase HIF-2α-regulated endogenous erythropoietin levels in patients with chronic kidney disease suffering from renal anemia^33^.

GeNorm analysis revealed that *B2M* and *YWHAZ* are among the three best performing reference genes identified in each of three biological replicates (Table 1). When analyzing the expression stability with the NormFinder algorithm^31^ both *B2M* and *YWHAZ* are consistently identified as the most stable reference genes to address the effect of hypoxia on melanoma cells (Table 2). B2M is part of the MHC class I molecules, which is present on almost all cells. In accordance with our results B2M was found to be stably expressed in hypoxic cultured human chondrocytes and bladder cancer cells^9,34^. In contrast B2M expression was found to be significantly altered in hypoxic prostate cancer cells^35^. YWHAZ is a central hub protein involved in many signal transduction pathways and plays a key role in tumor progression^36^. Contrary to our results, two studies systematically evaluating stability of internal reference genes for qPCR analysis of human neural stem cells preconditioned with hypoxia, and chronically hypoxic rat heart, identified *YWHAZ* as one of the least stable reference genes, underlining the need for proper validation of reference genes in every experimental setup^12,37,38^.

Our analysis showed that *ACTB* is the least stable reference gene in two out of three biological replicates, which is in accordance with other studies^9,34,39^. Hypoxic cells frequently undergo EMT, where differentiated epithelial cells are converted into poorly differentiated migratory and invasive mesenchymal cells^18,40^. This comprehends a profound remodelling of the cytoskeleton, which includes an altered expression of ACTB. Contradictory findings also exist, with *ACTB* observed to be stably expressed in some breast and prostate cancer cell lines under hypoxic conditions^7,35^. Despite varying *ACTB* mRNA levels following hypoxia in our study, normalization of CYGB protein levels in immunoblotting experiments was performed with stable levels of ACTB. Our data are in broad agreement with those of Staudacher and colleagues showing that low oxygen levels lead to an increase in untranslated *ACTB* levels, however only weakly impacting its protein expression which remains stable^41^. Collectively these observations highlight that gene expression stability under hypoxic conditions is strongly dependent on the origin of cells/tissues.

Our results indicate that after 24 h of environmentally and chemically induced hypoxia, *CYGB* mRNA levels were significantly upregulated in A375 cells, ranging from a four- to six-fold increase as compared to the normoxic condition (Fig. 2). In Malme-3M, *CYGB* was only slightly upregulated (Fig. 3). The lower response of Malme-3M cells to a hypoxic environment can partly be explained by the difference in intrinsic *CYGB* levels. Indeed, under normoxic conditions, we observed that *CYGB* mRNA levels were more than 800-fold higher in Malme-3M as compared to A375 cells. Moreover, when comparing the protein levels, we noticed strong differences between both cell lines with a 50-fold higher expression in Malme-3M as compared to A375 cells (data not shown). These data are consistent with previously reported results for different melanoma cell lines, G361, P22, C32TG, highly expressing *CYGB* (100- to 220-fold more than A375 cells) and only showing a slight or no induction under hypoxic conditions^21^.

In line with our data, several studies reported that *CYGB* is upregulated under strong hypoxic conditions in Hep3B, renal clear cell carcinoma (RCC4), transformed human bronchial epithelial cells (BEAS-2B), human cervix carcinoma (HeLa) and murine derived hippocampal neurons (HN33) cells^28,42,43^. Furthermore, some of these studies suggested the involvement of HIF-1α in the hypoxic regulation of *CYGB*^28,42^. This prompted us to further explore the molecular mechanism responsible for hypoxia-inducible *CYGB*, and specifically the contribution of HIF-1α and HIF-2α in A375 cells. Surprisingly, our results showed that only overexpression of HIF-2α induced *CYGB* promoter-driven luciferase activity, which was confirmed on protein level, whereas HIF-1α did not result in any detectable regulation. Additionally, these results were validated in the non-melanoma cell line Hep3B, even though a weak upregulation could be observed in the luciferase experiments upon HIF-1α overexpression. HIF-1α and HIF-2α have the same DNA-binding consensus sequence (5′-RCGTG-3′), however, cell type, duration, type of stimulation and culture conditions were reported to influence HIF-1α versus HIF-2α -mediated transcription^44–46^. Moreover, by overexpressing constitutively active HIF-1α and HIF-2α (i.e. with mutated proline residues) in primary endothelial cells, Downes and colleagues demonstrated that both HIF-α isoforms share more than 300 genes^44^. Furthermore, Smythies and co-workers showed that cell-specific gene induction by HIF-1α or HIF-2α arises by recruitment and association with other transcription factors that are enriched at HIF-1α or HIF-2α binding sites^47^. Therefore, it is conceivable that in Hep3B and A375 cells, HIF-2α, rather than HIF-1α, by recruitment and binding of other transcription factors, positively regulates *CYGB* under hypoxic conditions in a cell type specific way.

Throughout our study we applied 21% incubator O_2_ conditions and referred to this as normoxia. Although widely applied in physiological terms, these conditions are rather hyperoxic as not even lung alveolar cells are ever exposed to 21% O_2_. Because the cellular O_2_-sensing system is self-adaptive^48^, the absolute pO_2_ levels in the cellular microenvironment remain unknown^49^. In fact, hypoxia rather refers to a temporal than a spatial condition. Therefore, every decrease in pO_2_ leading to a biological effect, like a transient increase in HIFα protein stability, can be termed hypoxia^45^. For routine experimental work, it is broadly acceptable to compare at least two O_2_ concentrations that are sufficiently different from each other to cause specific biological effects while not affecting general cell viability^45^.

A possible limitation of our study is the use of UV/Vis spectra-based determination of RNA concentration. Ideally a specific fluorescent dye selectively binding RNA should be employed for the sensitive and accurate quantification of RNA^50^. On the other hand, UV/Vis spectrophotometry enables the simultaneous assessment of RNA purity, a factor contributing to potential variability in reference gene expression stability^51^.

In conclusion, our results underline the importance of selecting and validating an appropriate set of reference genes for gene expression analysis using real-time qPCR depending on cell type and experimental conditions. In particular, we have established that in two melanoma cell lines, Malme-3M and A375, *B2M* and *YWHAZ* are the most optimal genes to be used under experimentally-induced hypoxic conditions. Moreover, we have demonstrated that in A375 cells *CYGB* is HIF-2α-dependently regulated. The presented approach of normalizing hypoxia-inducible *CYGB* gene expression will be of major interest for further studies focussing on the importance and functional implications of hypoxic CYGB regulation and how this may impact melanoma cell survival, growth and spreading.

## Methods

### Cell culture

Human Malme-3M (ATCC HTB-64) melanoma cells were maintained in Roswell Park Memorial Institute (RPMI) 1640 medium (Gibco, Life Technologies), containing L-glutamine, supplemented with 10% heat-inactivated fetal bovine serum (FBS) (Gibco, Life Technologies) and 1% Penicillin/Streptomycin (10,000 Units/mL P; 10,000 μg/mL S; Gibco, Life Technologies). Human A375 (ATCC CRL-1619) cells were maintained in Dulbecco’s Minimum Essential Media (DMEM) (Gibco, Life Technologies), containing L-Glutamine, supplemented with 10% heat-inactivated FBS and 1% Penicillin/Streptomycin (10,000 Units/mL P; 10,000 μg/mL S; Gibco, Life Technologies). Both cell lines were incubated in a humidified 5% CO_2_ atmosphere (normoxia) at 37 °C and were routinely subcultured after trypsinization. For the hypoxic experiments 3.5 × 10^5^ (RNA extraction) or 2.5 × 10^6^ (protein extraction) A375 and Malme-3M cells were seeded out in 6-well plates or 100 mm culture dishes. The subsequent day hypoxia experiments were carried out at 0.2% O_2_ and 5% CO_2_ in a gas-controlled glove box (InvivO2 400, Ruskinn Technologies). Additionally, cells were treated with 100 μM roxadustat (FG-4592) (Sigma-Aldrich), or an equal amount of dimethyl sulfoxide (DMSO) as a vehicle control.

### RNA extraction, purification and cDNA conversion

RNA extraction and purification from A375 and Malme-3M cells cultured under normoxic or hypoxic conditions was performed using a RNeasy Mini Kit (QIAGEN) according to the manufacturer’s instructions. RNA concentration and purity were measured with an Implen NanoPhotometer^®^ N50 UV/Vis NanoVolume spectrophotometer (Implen). cDNA was reverse transcribed using PrimeScript™ RT Reagent Kit (Takara) according to the manufacturer’s protocol.

### Real-time quantitative PCR

Amplification of cDNA and subsequent quantification was performed on a CFX96 C1000 (BioRad) using a KAPA SYBR^®^ FAST qPCR reagent (Sigma-Aldrich). All PCR reactions were performed in duplicate for biological replicates with an inter-run calibrator (IRC) to detect and remove inter-run variation between the different mRNA quantification runs. The following conditions were used during PCR: 95 °C for 10 min and 40 cycles of: 95 °C for 15 s; 60 °C for 1 min. A list of the selected reference genes is given in Table 3. The following candidate reference genes were assessed: *ACTB*, *UBC*, *HMBS*, *SDHA*, *HPRT1*, *TBP*, *B2M* and *YHWAZ*. We also analysed *CAIX*, *GLUT1* and *PHD2* as established hypoxic control genes to monitor the efficacy of the hypoxia response. All primers were manufactured and provided by Eurogentec or Microsynth. Table 4 contains primer sequences, amplicon sizes and amplification efficiencies. Reaction efficiencies of PCR assays were derived from standard curves that were generated using serial dilutions of the corresponding cDNA. Amplification efficiency is determined using the formula 10^−1/slope^. For the actual calculations, the base of the exponential amplification function is used (e.g. 1.94 means 94% efficiency). Amplification efficiencies were subsequently used to transform the raw threshold cycle (C_t_) values to relative quantities by qBase software (version 3.2)^52^.

**Table 3:** List of candidate reference genes.

Gene symbol	Gene name	GeneID
*ACTB*	β-Actin	60
*B2M*	β-2 microglubulin	567
*HPRT-1*	Hypoxanthine phosphoribosyltransferase 1	3251
*SDHA*	Succinate dehydrogenase complex flavoprotein subunit A	6389
*UBC*	Ubiquitin C	7316
*YWHAZ*	Tyrosine 3-monooxygenase/tryptophan 5-monooxygenase activation protein zeta	7534
*TBP*	TATA-box binding protein	6908
*HMBS*	Hydroxymethylbilane synthase	3145

**Table 4:** Primer sequences, amplification efficiencies and amplicon sizes for candidate normalization genes and target genes.

Gene symbol	Forward primer	Reverse primer	Amplification efficiency	Amplicon size
**Reference gene**				
*ACTB*	AAAGACCTGTACGCCAACAC	GTCATACTCCTGCTTGCTGAT	1.94	219
*B2M*	TGCTGTCTCCATGTTTGATGTATCT	TCTCTGCTCCCCACCTCTAAGT	2.04	86
*HPRT-1*	TGACACTGGCAAAACAATGCA	GGTCCTTTTCACCAGCAAGCT	1.95	94
*SDHA*	GGAAGCATAAGAACATCGGAACTG	CTGATTTTCCCACAACCTTCTTGC	2.06	110
*UBC*	ATTTGGGTCGCGGTTCTTG	TGCCTTGACATTCTCGATGGT	2.03	133
*YWHAZ*	ACTTTTGGTACATTGTGGCTTCAA	CCGCCAGGACAAACCAGTAT	2.03	94
*TBP*	TGCACAGGAGCCAAGAGTGAA	CACATCACAGCTCCCCACCA	2.08	132
*HMBS*	AAGTGCGAGCCAAGGACCAG	TTACGAGCAGTGATGCCTACCAAC	1.93	298
**Target gene**				
*CYGB*	CTCTATGCCAACTGCGAG	AACTGGCTGAAGTACTGCTTG	2.04	89
*PHD2*	GAAAGCCATGGTTGCTTGTT	TTGCCTTCTGGAAAAATTCG	2.01	162
*GLUT1*	TCACTGTGCTCCTGGTTCTG	CCTGTGCTGAGAGATCC	1.98	230
*CAIX*	GGGTGTCATCTGGACTGTGTT	CTTCTGTGCTGCCTTCTCATC	1.89	309

Amplification efficiency is determined using the formula 10^−1/slope^.

For the actual calculations, the base of the exponential amplification function is used (e.g. 1.94 means 94% amplification efficiency).

### Analysis of gene expression stability by RT-qPCR

The stability of the reference genes expression was evaluated by the geNorm algorithm. GeNorm analyses the stability of reference genes transcripts taking into account the expression stability value (*M*)^12^. This stability value is calculated for each gene of a panel of candidate reference genes based on pairwise variation analysis. Moreover, lower values of *M* correspond to higher gene expression stability. Furthermore, geNorm is also capable to determine the ideal number of reference genes needed for accurate normalization.

### Protein extraction and quantification

Lysis buffer, containing 10 mM Tris HCl (pH 8), 1 mM EDTA, 400 mM NaCl, 1% NP-40 and protease inhibitors (Sigma-Aldrich) was used to lyse cells as described before^53^. Lysed cells were placed on a rotating arm at 4 °C for 30 min to allow optimal performance of the lysis buffer. The suspension was subsequently sonicated for 1 min at 60 Hz to degrade any potential formed DNA-aggregates. Finally, samples were centrifuged at 10,000 g for 15 min and the protein-containing supernatant was collected. Protein concentrations were determined using the Bradford Dye Reagent (Chemie Brunschwig).

### Immunoblotting

Extracted proteins for immune-based western blotting were first separated, according to molecular weight, using sodium dodecyl sulphate polyacrylamide gel-electrophoresis (SDS-PAGE) gels, followed by electrotransfer to nitrocellulose membranes (Amersham Hybond-ECL, GE Healthcare) as described before^54,55^. Equal amounts of protein and volume were loaded onto a 7.5% polyacrylamide gel for HIF-1α and HIF-2α, and 15% polyacrylamide gel for CYGB. Membranes were blocked in TBS-T (Tris-buffered Saline; 0.1% Tween-20), containing 5% non-fat dry milk, for 1 h at room temperature. After blocking, membranes were incubated overnight at 4 °C with primary antibodies (anti-HIF-1α, BD Transduction Laboratories, 610958; anti-HIF-2α, Bethyl, A700-003; anti-GFP, Proteintech, 50430-2-AP-150UL; anti-β-actin, Sigma, SP124). The following day, membranes were washed with TBST-T, and incubated during 1 h with horseradish-conjugated secondary antibodies (anti-mouse IgG HRP, Sigma, GENA931-1ML, anti-rabbit IgG HRP, Sigma, GENA934-1ML). The signal was revealed using ECL Prime (Amersham, GERPN2232) on a C-DiGit^®^ Western blot scanner (LI-COR Biosciences), and exported and quantified using Image Studio™ program (LI-COR Biosciences). Uncropped immunoblots are provided in Supplemental Fig. S3.

### Luciferase reporter assays

*CYGB* promoter construct generation was described before^25^. 3 × 10^5^ Hep3B or 3.5 × 10^5^ A375 cells were transiently transfected with 300 ng reporter plasmid and YFP-HIF-1α or YFP-HIF-2α as indicated, in a six-well format using JetOptimus (Polyplus). To control for differences in transfection efficiency and extract preparation, 25 ng pRL-SV40 *Renilla* luciferase reporter vector (Promega) was co-transfected. Cultures were evenly split onto 12-well plates 24 h after transfection. Luciferase activities of triplicate wells were determined using the Dual Luciferase Reporter Assay System (Promega) as described before^56,57^. Reporter activities were expressed as relative firefly/*Renilla* luciferase activities (R.L.U.). All reporter gene assays were performed four to eight times independently.

### Statistical analysis

All values in the figures are presented as mean ± standard error of the mean (SEM). Differences in means between two groups were analyzed with unpaired 2-tailed Student’s t-test and those among multiple groups with one-way ANOVA followed by Tukey posthoc test. All statistics were performed with GraphPad Prism software 7.05. Values of *p* ≤ 0.05 were considered statistically significant.

## Supplementary Information

Supplementary Information.

## Data Availability

All data generated and analysed in this study are available from the corresponding author upon request.
