# Supplementary material for: A reliable set of reference genes to normalize oxygen-dependent cytoglobin gene expression levels in melanoma
Source: Sci Rep. 2021 May 25;11:10879. doi: 10.1038/s41598-021-90284-6 (PMC8149659; doi:10.1038/s41598-021-90284-6)
Supplement: Supplementary file 1 — Supplementary Information. [file 41598_2021_90284_MOESM1_ESM.pdf]

Supplemental Information

A reliable set of reference genes to normalize oxygen-dependent cytoglobin gene expression levels in melanoma.

Joey De Backer [1,2,$], Darko Maric [2,$], Matthias Bosman [1], Sylvia Dewilde [1] and David Hoogewijs [2,*].

[1]Research Group PPES, Department of Biomedical Sciences, University of Antwerp, Belgium.
[2]Section of Medicine, Department of Endocrinology, Metabolism and Cardiovascular System, University of Fribourg, Switzerland
[$] equal contribution

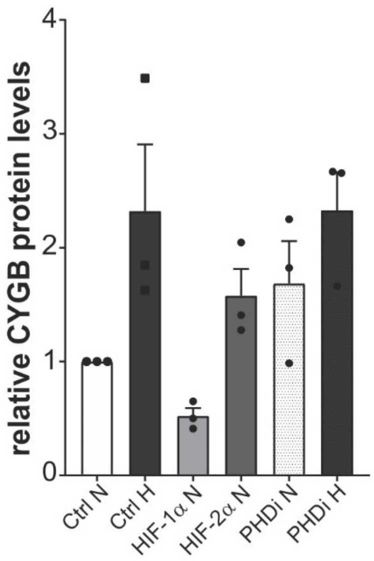

Supplemental Figure S1. Quantification of protein levels of Figure 5A. Quantification of CYGB protein levels from 3 independent replicates (mean ± S.E.M; n = 3) of A375 cells after 48 hours cultured under normoxic (N) or hypoxic (H) (0.1% O_2) conditions, in the presence of overexpressed YFP-HIF-1α or YFP-HIF-2α, and upon treatment with 4 mM PHD inhibitor (PHDi).

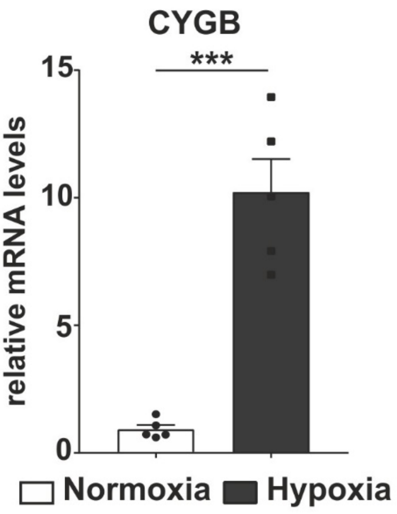

Supplemental Figure S2. CYGB mRNA expression levels are hypoxia-inducible in Hep3B cells. CYGB expression levels measured by qPCR in Hep3B cells cultured for 24 hours under normoxic and hypoxic conditions. qPCR values were normalized to *B2M* and *YWHAZ* (mean ± S.E.M; n = 5). Individual values of replicates are depicted as black dots. Student's t-test (***$p \leq 0.001$).

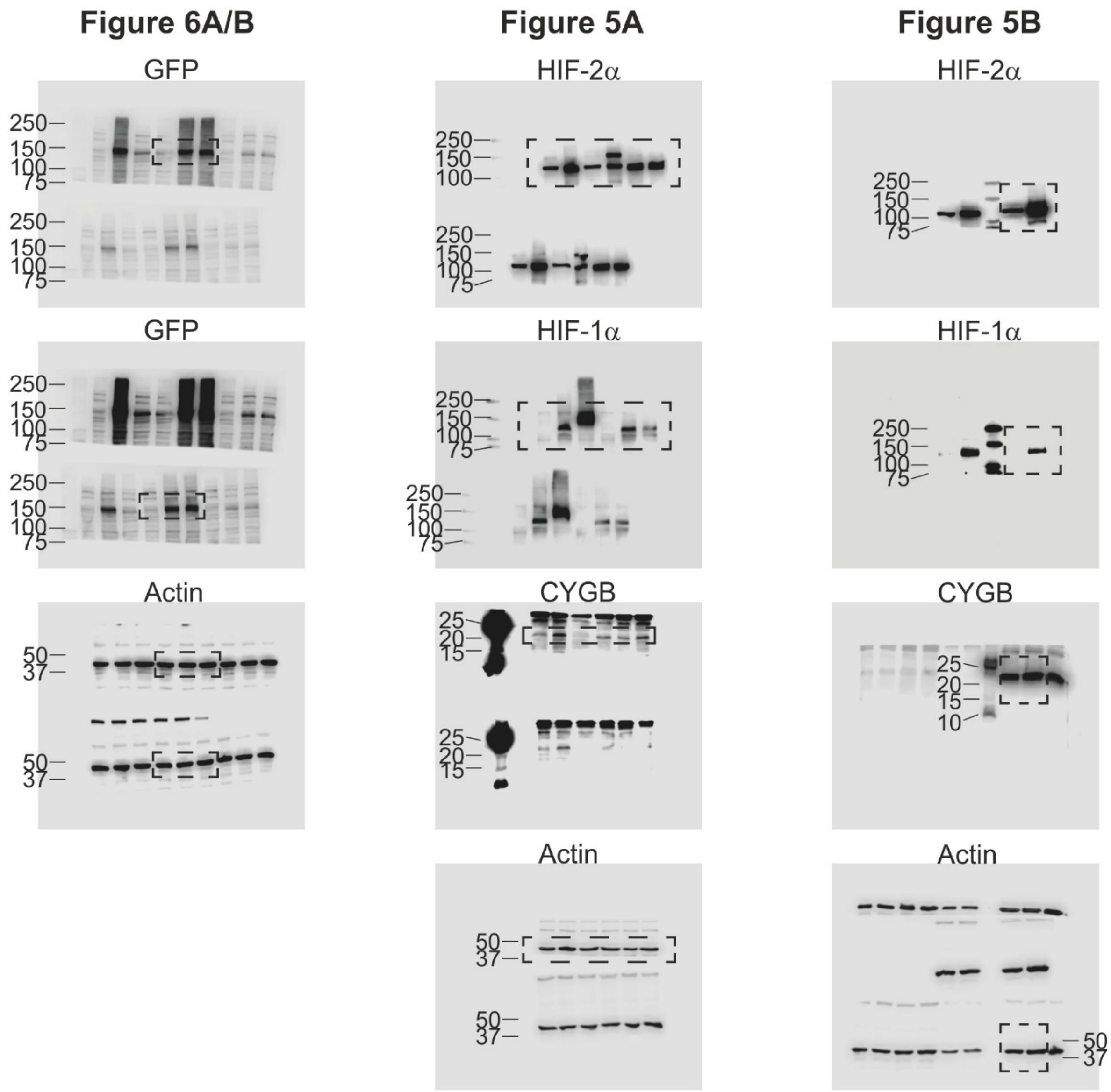

Supplemental Figure S3. Original full-length versions of immunoblots shown in Figures 5 and 6. Uncropped immunoblots with indicated antibodies are displayed.
